# “I may be essential but someone has to look after my kids”: women physicians and COVID-19

**DOI:** 10.17269/s41997-021-00595-4

**Published:** 2021-12-17

**Authors:** Julia Smith, Lina Abouzaid, Joy Masuhara, Salima Noormohamed, Neli Remo, Lynn Straatman

**Affiliations:** 1 Faculty of Health Sciences, Simon Fraser University, 11806 Blusson Hall, 8888 University Dr., Burnaby, BC V5A 1S6 Canada; 2 Vancouver Physician Staff Association, Vancouver, BC Canada; 3 Department of Family Practice, Faculty of Medicine, University of British Columbia, Vancouver, BC Canada; 4 Culture and Environment Working Group, VCH Physician Diversity Equity and Inclusion Committee, Vancouver, BC Canada; 5 Physician Engagement & Program Development, Vancouver Coastal Health, Vancouver, BC Canada; 6 Physician Engagement, Vancouver Coastal Health, Vancouver, BC Canada; 7 Division of Internal Medicine, Faculty of Medicine, University of British Columbia, Vancouver, BC Canada

**Keywords:** COVID-19, Women, Gender, Physicians, Health systems, Leadership, COVID-19, femmes, genre, médecins, systèmes de santé, leadership

## Abstract

**Objectives:**

This paper analyzes results from focus groups held with women physicians in British Columbia which explored questions around how gender norms and roles influenced their experiences during COVID-19.

**Methods:**

Four virtual focus groups were organized between July and September 2020. Participants (*n* = 27) were voluntarily recruited. Data were analyzed using applied thematic analysis.

**Results:**

In addition to the COVID-19-related changes experienced across the profession, women physicians faced distinct challenges related to an increase in unpaid care responsibilities, and often felt excluded from, and occasionally dismissed by, leadership. Women leaders often felt their contributions were unrecognized and undervalued. Participants drew strength from other women leaders, peer networks, and professional support, but these strategies were limited by unpaid care and emotional labour demands, which were identified as increasing risk of burnout.

**Discussion:**

Even though women physicians hold a degree of relative privilege, unpaid care work and gender norms contribute to distinct secondary effects of COVID-19. Women physicians link these to pre-pandemic assumptions (within families and communities) that women would absorb care deficits at their own cost. Health system leadership continues to reflect a masculine normative experience wherein the personal and professional are separated, and which devalues the emotional labour often associated with feminine leadership. The strategies participants employed to address negative impacts, while demonstrating resourcefulness and peer support, reflect individualistic responses to social-structural challenges. There is a need for greater recognition of women’s contributions at home and work, increased representation in decision-making, and practical supports such as childcare and counselling.

## Résumé

**Objectifs:**

Dans cet article, nous analysons les résultats de groupes thématiques tenus avec des femmes médecins en Colombie-Britannique pour explorer des questions sur l’influence des normes et des rôles liés au genre sur les expériences vécues durant la COVID-19.

**Méthode:**

Quatre groupes thématiques virtuels ont été organisés entre juillet et septembre 2020. Les participantes (*n* = 27) ont été recrutées parmi des volontaires. Les données ont fait l’objet d’une analyse thématique appliquée.

**Résultats:**

En plus des changements liés à la COVID-19 vécus dans toute la profession, les femmes médecins ont connu des difficultés particulières liées à l’augmentation de leurs responsabilités domestiques non rémunérées et se sont souvent senties exclues, et parfois rejetées, par la haute direction. Les dirigeantes ont souvent senti que leurs contributions étaient non reconnues et sous-estimées. Les participantes se sont appuyées sur d’autres dirigeantes et sur leurs réseaux de pairs et de soutien professionnel, mais ces stratégies ont été limitées par les exigences de leur travail domestique non rémunéré et de leur labeur émotionnel, qui sont reconnues comme faisant augmenter le risque d’épuisement professionnel.

**Discussion:**

Bien que les femmes médecins soient relativement privilégiées, le travail domestique non rémunéré et les normes liées au genre ont contribué à des effets secondaires de la COVID-19 qui leur sont particuliers. Les femmes médecins expliquent ces effets par l’hypothèse, qui prévalait déjà avant la pandémie (au sein des familles et des communautés), que les femmes combleraient les lacunes dans les soins à leurs propres dépens. Les dirigeants des systèmes de santé continuent de refléter une expérience masculine normative selon laquelle la vie personnelle et la vie professionnelle sont séparées, et qui dévalorise le labeur émotionnel souvent associé au leadership féminin. Bien qu’elles fassent preuve d’ingéniosité et d’entraide, les stratégies employées par les participantes pour aborder les impacts négatifs reflètent des réponses individualistes à des difficultés sociostructurelles. Il est nécessaire de mieux reconnaître les contributions des femmes à la maison et au travail, d’accroître leur représentation dans la prise de décisions et de renforcer les mesures d’aide pratiques comme les services de garde et le counseling.

## Introduction

There is a growing literature on healthcare worker experiences during COVID-19 and the effects of the pandemic on well-being and career development, including studies which disaggregated findings by sex and/or gender (Kurt et al., 2020; Kelker et al., 2020). Many of these studies demonstrate higher rates of burnout, anxiety, and financial loss among women healthcare workers as compared with their male counterparts, but provide little detail as to the drivers of these inequities (Delaney et al., 2021; López-Atanes et al., 2021). Furthermore, there is little empirical research on gender differences among healthcare workers during emergencies that focuses on the experiences of physicians. As physicians are one of the few healthcare professions where the majority identify as men, with just 43% of physicians in Canada identifying as women, there is a need for distinct gender analysis (Cohen & Kiran, 2020). A number of commentaries have touched on the unique experiences of women physicians during COVID. Brubaker (2020) notes that the profession continues to be characterized by a persistent “work–life imbalance” that particularly disadvantages women, the effects of which, she predicts, will be exacerbated by the pandemic. Similarly, Jones et al. (2020) discuss how the pandemic may increase unpaid care work for women physicians leading to reduced paid work. However, there is a lack of evidence of not only the effects of COVID-19 on women physicians specifically, but also on the underlying drivers of gender inequities within the profession.

This paper aims to contribute to filling this gap by analyzing findings from four focus groups organized by the Vancouver Coastal Health Physician Diversity Equity and Inclusion Committee (DEIC) and Vancouver Physicians’ Staff Association (VPSA), held in the Vancouver Coastal Health Authority (VCH) of British Columbia. The primary purpose of the focus groups was to inform advocacy activities for the DEIC and VPSA, with the secondary focus being to explore research questions around what is distinct or unique about the experiences of and challenges faced by women physicians during COVID-19. Our findings illuminate two key challenges faced by women physicians; the first related to the heightened tension between unpaid care and professional obligations and the second related to gendered leadership dynamics. While women physicians developed individual and peer support strategies in response to these challenges, these were restricted by structural inequities, many of which pre-date COVID-19.

### Gender-based analysis

We explore these themes through a gender-based analysis lens, recognizing that gender roles and norms structure both individual experiences and health systems. Gender norms refer to the often unspoken rules that govern the attributes and roles that are valued and considered acceptable for men, women, and gender-diverse individuals: “Norms are embedded in institutions, defining who occupies leadership positions, whose contributions are valued, and whose needs are accommodated” (Morgan et al., 2016). Gender-based analysis seeks to make these norms explicit and identify gender bias within policies and processes. Gender bias encompasses more than just personal perception, it also includes a blindness to the policies and structures “that operate in favour of men as a gender, and against women as a gender” (Elson, 1993). Gender roles include tasks and behaviour deemed appropriate and expected of a particular gender both at work and at home. Feminist economics and the care economy literature have demonstrated how gender roles shape who does, and the conditions of, care work, with women around the world doing the majority of unpaid care work. Responsibility for unpaid care work, such as child and elder care, means that when paid care services are not accessible, women absorb care burdens at their own, often unrecognized, costs—such as reduced employment and empowerment opportunities (Folbre, 2006). This has proven particular true during health crises, such as COVID-19 (Hupkau & Petrongolo, 2020).

Care work includes both physical and emotional acts of caring, including emotional labour, i.e., “the induction or suppression of feeling in order to sustain an outward appearance that produces in others a sense of being cared for in a convivial safe place” (Gray, 2010). Emotional labour has traditionally been identified with women’s work and the role of the mother in the family. The portrayal of emotional labour as a “natural activity” results in its devaluation in cultural and economic terms (Gray, 2010). Such assumptions also result in lack of recognition for emotional labour conducted in professional settings. Health systems research has documented how women healthcare workers take on more emotional labour in terms of patient care, partly due to gender norms and partly due to the gendered composition of the healthcare workforce (with more women being in positions such as nursing that require high levels of personal contact with patients) (Elliott, 2017). There is less research on gendered differences related to emotional labour in terms of peer support and leadership.

There is however substantial research that demonstrates how health systems reproduce gender inequities, both in terms of patient care and among healthcare workers (Morgan et al., 2016). Among physicians, this is most evident in not only the greater proportion of men than women, but also the persistent gender wage gap and greater number of men in leadership positions (Cohen & Kiran, 2020). These inequities are attributed to a combination of factors, including discriminatory hiring procedures, women physicians’ unpaid care responsibilities, and metrics of success, such as research funding, that tend to favour men (Butkus et al., 2018). Leadership traits more often associated with men (for example, used more frequently in reference letters for male physicians), such as being assertive, are prized over those most often associated with women, such as being collaborative (Trix & Psenka, 2003; Turrentine et al., 2019). The analysis that follows recognizes health systems as gendered, positioning women physicians’ experiences within these broader structures of inequity.

## Methods

Focus group participants were recruited voluntarily via advertisements emailed from the DEIC and VPSA to all physicians working in VCH. Interested women physicians registered through an online portal and joined the focus groups through Zoom. Two focus groups were held in July (with 8 and 6 participants) and two were held in September (with 7 and 6 participants). All participants (*n* = 27) identified as women, representing a range of ethnicities, types of physicians, ages, and years of experience.

The limited sample size reflects the challenge of conducting research with essential workers during a public health crisis and is consistent with qualitative studies conducted in similar circumstances (see for example Erland & Dahl, 2017). Considering sample size and methods, the aim here is not to provide representative findings (which is why participants are not disaggregated by race, specialization, or other factors), but to analyze the perspectives of a group of women around a shared experience. We recognize this experience is partially shaped by geography and timing. VCH is an urban, centrally located health authority that experienced the second highest number of COVID-19 cases in the province. Focus groups were held during the initial months of the pandemic, often referred to as the first wave, when healthcare workers were adapting to new ways of working in a context of uncertainty.

Being unwilling to press frontline workers into adding to their schedules, we held as many focus groups as there was interest in, as opposed to aiming to reach saturation. Consequently, many findings point to further lines of inquiry, as opposed to conclusions. While recognizing these limitations, we contend that such qualitative research adds value in the documenting of lived experiences of specific groups and including meaningful inquiry in health research and policy discussions (Sallee & Flood, 2012).

Written informed consent information was sent to participants ahead of time by email and then verbally reviewed at the beginning of the focus groups. Information included why the DEIC and VPSA were conducting the focus group (i.e., to better understand women’s experiences and advocate for policy change), as well as the broader research goals. Participants provided consent for focus group data to be used for research purposes through a poll function at the beginning of each focus group. Participants, facilitators, and the authors were present in Zoom focus groups. Ethical approval was provided by the Office of Research Ethics at Simon Fraser University.

Focus groups were facilitated by women leadership coaches from the VCH People and Culture team, who had training in peer facilitation. Focus groups lasted an hour and included a combination of open discussions and pre-set questions, which were anonymously answered via typing or polls. Pre-set questions related to experiences during COVID-19, perceptions of gendered differences of experiences, impacts on personal and professional lives, and engagement in leadership and decision-making. Focus groups were not recorded as the facilitators felt recording might make participants uncomfortable and unwilling to share candidly. As the primary purpose of the focus groups was to inform DEIC and VPSA activities, with research being the secondary focus, participants’ comfort was prioritized. Instead of recording, two scribes (one a member of the DEIC and the other the academic researcher) took anonymized notes, including writing down direct quotations; typed and poll responses were saved. As data were anonymized at recording, quotes are identified by date of focus group (in the format of day.month.year).

Four of the authors of this paper were facilitators and one observed as a researcher. As the facilitators were members of the DEIC or VPSA, including two who were also women physicians, their personal experiences and perceptions of the issues under discussion necessarily influenced both the focus group process and data analysis. Such bias can be a strength in that it enables facilitators to identify nuances that may be missed by someone without lived experience expertise (Kamberelis & Dimitraidis, 2012). As an observer, the fifth author provided an external perspective, often seeking out clarity and questioning assumptions during the analysis.

Focus group notes were compared to ensure consistency and accuracy and, together with typed answers to questions, analyzed using applied thematic analysis (Guest et al., 2014). Three authors (two facilitators and the external researcher) first independently familiarized themselves with the data, constructing themes and subthemes through a combination of deductive (based on focus group questions) and inductive analysis (based on participant discussions), and then compared themes to co-develop a code book, which was shared with the other authors for further discussion and finalization. The final analysis resulted in top level themes reflecting the deductive coding, and sub-level themes being constructed from the data. For example, ‘double shift’ was a top-level theme developed from research questions about challenges faced by women physicians, but ‘unable to outsource’ and ‘kids always want their mom’ were derived inductively from contributions. The first author then used the code book to analyze the data in NVivo, with the analysis reviewed by co-authors.

## Results

### The double shift

**Fig. 1:**
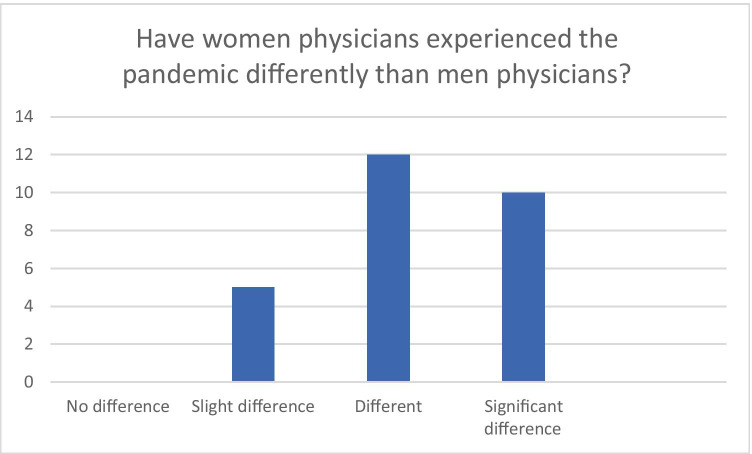
Results compiled from mentimeter polls in all four focus groups

Physicians discussed general impacts of the first wave of, or initial response to, COVID-19 felt across the profession, including stress related to transitioning to new ways of working in a context of constantly changing information regarding risk and, depending on speciality, financial uncertainty. They also noted women physicians experienced distinct effects, with many citing a key difference being the increased labour of “the double shift” of professional and care responsibilities that women physicians, more often than their male counterparts, assumed (Fig. 1).

Those mothers in heterosexual relationships noted that the responsibility to adapt childcare arrangements in response to COVID-19 related to school and daycare closures fell on them: “I have a young child at home and that has been the most challenging thing to deal with. The additional home responsibilities were daunting and that was in addition to the precarious work situation.” (06.07.20). While physicians had access to childcare for essential workers during initial closures from March to June 2020, the challenge of making new arrangements was exacerbated by lack of a coordinated emergency response within the childcare sector (it was up to providers to decide if they would continue to operate and at what capacity) and a pre-COVID extreme shortage of childcare in the Vancouver area, especially for parents engaged in shift work (Macdonald & Friendly, 2021). In particular, physicians who relied on in-home care, often laid off nannies (so they could access the government benefits and not risk infection which was perceived as higher due to the physicians’ work) and then had to seek out facility-based care for essential workers, which rarely provided flexibility for shift work. One mother noted her child’s centre only provided care from nine am to three pm, asking “who works until 3 pm?” (21.09.20).

Numerous focus group participants noted they, as opposed to the men in their families, were responsible for the care of elderly family members, stating “Daughters tend to take care of elders by default.” (07.07.20). Care responsibilities added an extra layer to the anxiety and stress physicians were facing: “Initially, there was a lot of effort invested in restructuring how we do our clinical work. That coupled with constant worry about elder parents who were ill during a time when the elderly were most at risk was challenging and a source of anxiety.” (07.07.20). Another participant similarly noted, “caring for patients virtually and the technological challenges in addition to health issues experienced by the extended family all added up to a very stressful period.” (06.07.20). Some had taken measures to distance themselves from elderly family members, but this was replaced by guilt related to unfulfilled care responsibilities: “You’re not just worried about yourself, but if you’re going to give it to others, like elderly parents.” (07.07.21).

Many women physicians had previously managed the double shift of care work by outsourcing tasks, but such services were interrupted during the initial months of lockdown: “Because I couldn’t outsource, my housekeeper couldn’t come, I had to do all of this. It was extremely frustrating how imbalanced it felt. It all came to me.” (06.07.20). It was felt that, in both families with and without children, women were expected to resolve increased care burdens. “If the cleaner can’t make it the mom just ends up doing it all.” (07.07.20).

Participants discussed why women were primarily responsible for unpaid care work. The feeling that “kids always want their mom” was reiterated by numerous participants in multiple focus groups. Women recognized their own participation in perpetuating gender norms, speaking of the challenge of delegating to others. One participant explained “My husband provided childcare when I was working however, because I was in the house, I kept having the inclination to shorten my workday, or to intervene to help my husband to take care of our child.” (06.07.20). Another asked, “I seem to need to be responsible for more things than men... where does that come from?” (29.09.20). Such discussions suggest that the heightened professional and care demands of the pandemic made explicit women’s internalized assumptions that they should be able to manage the double shift. This was further evidenced in attitudes towards risk of infection and the possible need to isolate. Mothers felt unable to leave their children: “I have children and not being able to isolate was a big worry for me.” (07.07.21). Participants shared anecdotes of men isolating in hotels, while women camped out in the garage or backyard, noting “Male colleagues were better able to isolate for the week they were on COVID ward. Women wanted to isolate within home because children are going to want their moms. Most of the women didn’t feel they could up and leave.” (07.07.21).

While such quotes position women physicians as imposing standards of family care upon themselves, participants noted that assumptions regarding who is responsible for unpaid care also originated from social and health system responses. For example, a mother noted she was interrupted in the operating room by a call from her child’s school because her child had developed possible COVID-19 symptoms, even though the school also had the father’s number. Another participant similarly noted, “My kids had to isolate and were sent home. The school calls me at one pm and assumes that I should just go home and take care of the kids, even though my husband is far more flexible with his schedule. Why didn’t they call him?”(21.09.20).

When physicians were unable to meet both care and professional demands, they incurred costs, including having to reduce paid work hours and research productivity. One participant noted she had just that day given up a call, which a male colleague took on, because her kids were sick. Another mentioned that while physicians were provided with quarantine pay through Doctors of BC if they contracted COVID-19, this was not provided if they could not work because their kids had to isolate, noting “I may be essential, but someone has to look after my kids.” (21.09.20). Physicians also reported losing sleep and feeling more anxious due to increased care work, noting declining mental wellness was widespread among peers: “[I was] at a committee meeting with family physicians and a lot were getting very fatigued and almost burnt out, if not burnt out, particularly the ones with children,” (29.09.20) and “I’ve seen others juggling family life, virtual care, then more things are opening up, it’s scary. Requirements keep changing, people are fatigued.” (29.09.20).

### Gendered leadership dynamics

Many participants felt that the challenges they faced as women physicians were not recognized by decision-makers, who were primarily men without similar care responsibilities: “Male leaders were making decisions, that had to be made quickly, but also had a stay-at-home wife, or children who were no longer dependent on them.” (29.09.20). Many participants indicated that the changes in scheduling, due to the COVID-19 response, did not take into consideration unpaid care responsibilities, with one participant noting, “The assumption is that the mother of the child will stay home” (21.09.20), and another stating, “They assume there is this model where there is someone to look after kids.” (21.09.20). A further participant noted that even when care responsibilities were recognized, the financial conflict that care work created for women physicians was not:

In my department it was expressed that if women wanted to take off time to take care of kids, people, meaning men, would take their call for them. People thought they were really well meaning, and they were even evolved, that they were men of a new age that they were doing this. Then it was pointed out to them that they were just asking women, that they assumed women don’t need an income. (21.09.20).

It was felt that women physicians’ need to both work and care for family was not recognized by a system that assumed physicians had the choice to prioritize one or the other.

Participants described contrasting ‘masculine’ and ‘feminine’ leadership styles in generalizable terms, often reflecting gendered assumptions noted in the leadership literature (Blake-Beard et al., 2020), but also grounded in their own experiences. Participants described the mostly male leadership as taking “command and control” style approaches during the first few months of the pandemic: “Men are making more decisions now with less process or consultation, they are more directive and dictatorial. Men are advancing their careers and using a more command and control style.” (21.09.20). While it was recognized that, in the context of an unfolding emergency, decisions often had to be made rapidly, lack of consultation also made decision-making more exclusive: “Men have become more directive. They’re using COVID to avoid consultation. They seem to just proceed and decide.” (29.09.20).

Some physicians felt that the COVID-19 response included a return to patriarchal attitudes, describing instances of sexist comments and talking down to women: “The old school, traditional male traits became very prominent during this time. Men in leadership roles become more intolerant and abusive verbally, and overall difficult to deal with.” (07.07.20). One participant felt such behaviour reflected unhealthy expressions of stress: “Because everyone was stressed everyone was anxious, I saw a regression to intolerance. Old school man behaviour was heightened during COVID, the mansplaining, the talking down to women.” (07.07.20). Another felt the emergency response was used to excuse such behaviour: “[COVID] allowed people to just assume and make statements like that. They felt like this before but now its ok to say.” (21.09.20). It was recognized that such behaviour did not just affect women, but also younger men and physicians from ethnic and racial minorities. Such abuse discouraged some women from participating in decision-making: “It has affected me emotionally that a lot of that abuse was turned towards me, to the extent that I didn’t want to attend meetings with those leaders any longer.” (07.07.20).

Conversely, focus group participants perceived women leaders as being more consultative and caring. Many participants noted they had received encouragement and support from women leaders who, despite the urgency of the emergency response, took time to ask about mental health, overall wellness, and family life: “I had to work from home and the response of my female colleagues in leadership roles was more compassionate and supportive during this time. Women leaders tended to ask more ‘how are you today?’ and ‘How are things at home?’” (06.07.20), and “A non-physician woman manager sent out an email telling everyone to give themselves a break and take some time off – being reminded of that helped.” (06.07.20).

Those participants who had engaged in leadership activities felt their efforts to create a caring work environment were undervalued, critiqued, and costly: “A colleague said to me ‘leave your heart at home’, women do get more emotionally involved, and it can be detrimental to our health. For those reasons it does put people off from stepping up to leadership roles.” (07.07.20). Participants spoke of feeling both overburdened by the need to support others during the crisis and the sense that their emotional labour was undervalued: “I am told that ‘I care too much’. And it’s true, I care.... I don’t know if I will be able to continue to be a leader for too long. The mental well-being toll is great.” (07.07.20). This combination was identified as increasing risk of burnout: “I worry that as women physicians are the ones who step up they will be the ones that have the post-traumatic stress, which will deplete women in leadership positions. It will set us back.” (07.07.20).

While women leaders felt critiqued for “caring too much”, a number also felt they were not given credit for their formal professional contributions. One participant recounted an instance where she was assigned to compile some documentation early in the outbreak, but that when it came time to present, men physicians got credit for her work. Another responded, “I echo the experience of seeing women doing the work and men taking the credit. Usually through having a leadership position and presenting the work.” (21.07.20). Others noted, “Seems men have the face time of the department, yet women do the work behind the scenes.” (21.07.20).

### Sources of strength

Women drew strength from the inspiring examples of women in leadership positions: “I have been so inspired by female leadership we have seen at this time” (21.09.20), and “Some people who weren’t traditionally visible have been which has been great to see.” (21.09.20). Women physicians also drew strength from peer support networks that connected through email chains and virtual group chats. One participant noted, “The silver lining of this pandemic was that it has created opportunities for collegiality – many virtual groups were set up across the country and this has encouraged women to interact with each other and support each other. Predominantly, women joined these groups.” (06.07.20). And another participant described how her peer group used humour to mitigate stress: “People started sharing jokes within the group... Someone compiled the jokes and had them printed on toilet paper. It has been amazing.” (29.07.20).

Many participants also noted that the shift towards more virtual work had benefits in terms of managing unpaid care and paid work. Being able to work from home and to attend meetings virtually, and having reduced travel were seen as advantageous for women who had previously found attending conferences and meetings difficult due to responsibilities at home: “It feels like a new phase of life, where things can be done differently, and people have to find new ways to become a leader. It has given faith that maybe it can be done without all the expectations for putting in face time, conferences, etc.” (21.09.20).

Numerous participants had benefited from mindfulness workshops, which were offered to all physicians, and commented on how they noted most of the participants were women. Others had accessed counselling services, and many noted that physical exercise and “me time” were essential to their overall well-being. Many also noted finding such time was challenging in the context of increased unpaid care work and keeping up with rapidly changing protocols. Women offered ideas on how these barriers could be overcome, ranging from having flexible childcare options on site, to intentionally inclusive leadership structures and leadership training specifically for women. It was also felt that mental health supports need to be provided within hospital settings, with some targeted at men who expressed stress in harmful ways. As one participant put it, “We all need psychological PPE.” (29.09.20).

## Discussion

The perspectives of focus group participants demonstrate that they never simply experienced the initial months of the pandemic as physicians; they lived it as women with increased care responsibilities outside of the formal health systems and limited opportunities within it. Women physicians noted their experience of COVID-19 was affected in distinct and negative ways by an increased double shift, which they linked both to pre-pandemic assumptions (within families and communities) that women would absorb private care deficits at their own cost and to the lack of supportive health systems. Health system leadership was identified as continuing to reflect a masculine normative experience wherein the personal and professional can be separated or balanced. Lack of consideration for women’s experience and needs was intensified by lack of consultation during the emergency response. Women felt their leadership contributions were often overlooked or coopted, and the emotional support they provided undervalued. In response, women physicians sought inspiration and support from other women leaders and peers, drawing on wellness resources.

Research on the general Canadian population has shown that while men have taken on more unpaid care work in the context of COVID-19, women still continue to shoulder the majority (Shafer et al., 2020; Qian & Fuller, 2020). This paper indicates a similar trend. Even though women physicians hold a degree of relative privilege, participants describe being overwhelmed by care work in ways they felt male physicians were not. Care burdens stemmed from gender norms—perpetuated both by the respondents who felt an obligation to conform and by assumptions within the health system—and from economic structures, such as lack of flexibility in work schedules and limited access to childcare. For example, the gender pay gap combined with inaccessible childcare often results in women, as opposed to men, giving up paid work to conduct unpaid childcare (Grönlund & Magnusson, 2016). While the dynamics of the care economy predate COVID-19, the pandemic exacerbated care deficits.

Within the initial response, the “tyranny of the urgent”, which causes emergency responses to deprioritize consultation and equity goals, prioritized “command and control” style leadership, resulting in women physicians feeling unrepresented, and at times dismissed, by masculine dominated leadership (Smith, 2019). Unequal representation in medical leadership prior to the outbreak, and lack of consultation during it, combined with time limits imposed by the double shift reduce women physicians’ opportunities to communicate and respond to the particular challenges they faced, entrenching masculine bias in decision-making. More women, along with other equity-seeking groups, in health system leadership and emergency response could mitigate this bias—if such leadership is adequately supported and valued.

The experiences of women physician leaders described here are complex. The perception of women leaders as more caring than men may reflect personal internalized gender bias, in that women physicians are more likely than their male counterparts to be described with related terms such as compassionate, but participants’ examples suggest such perceptions were also based on personal experiences (Carnes et al., 2015). Previous research has found that women physicians in Canada felt greater competency in non-verbal communications and handling sensitive issues than their male counterparts during patient interactions (Lovell et al., 2009); results here suggest these skills are also employed in interactions with peers. Whether women take on emotional labour or fulfill more ‘caring’ leadership roles because of internalized stereotypes (i.e., they feel it is expected of them) or to fill a gap that is not being met by other leaders, or a combination of the two (and possible other factors), is not a debate that can be evidenced here. What is apparent is that women leaders were identified as contributing these leadership qualities within the COVID-19 response, and these contributions, while valued by other women participants and in the health literature (Mousa et al., 2021), were perceived as being either ignored or seen as weakness within the health system more broadly. Participants described being critiqued for “caring too much”, with caring positioned as a personal liability as opposed to an asset in the COVID-19 response. Such devaluing reflects stereotypes in terms of what type of leadership is celebrated (e.g., authoritative, decisive, and rational as opposed to collaborative, caring, and emotional) within the medical field in general, and during a crisis response in particular (Fontenot, 2012).

While previous studies have documented women healthcare workers’ increased risk of burnout and mental health challenges, our findings suggest unpaid care work at home and emotional labour at work are determinants exacerbated by lack of recognition and support within health systems. Currently, the strategies participants employed to address these threats, while demonstrating resourcefulness and peer support, reflect individualistic responses to social-structural challenges. As Tricco et al. (2021) write, “Multipronged interventions composed of a combination of structural and individual interventions... are needed to foster lasting and meaningful change... solutions must begin with recognition of the systemic nature of the problem.” Similarly, Mousa et al. (2021) note that organizational interventions, as opposed to those that place responsibility on the individual, are needed to develop health systems supportive of women leaders. Many of the approaches listed in both Tricco et al. (2021) and Mousa et al. (2021) can be adapted to emergency situations, and if implemented in non-emergency time may prevent the negative impacts documented here.

While the focus here is on the experiences of women, gender refers to how relationships and structures are shaped by norms, roles, and power dynamics across a range of factors. In particular, intersectional gender-based analysis recognizes the need to incorporate other identity factors such as race, ethnicity, and (dis)ability (Hankivsky, 2012). Further research might take a more nuanced analysis to consider the experiences of different groups of women physicians, and how gender norms and roles particularly shape men and gender-diverse individuals’ experiences of the pandemic. Based on four focus groups with women physicians in one health authority in British Columbia, these findings cannot be taken as representative of women physicians’ experiences, or of gender dynamics within the profession more broadly. Not only are women physicians a diverse group, but their experiences will differ geographically and culturally. Consequently, this qualitative data and analysis is more illustrative than conclusive, sharing the perspectives of a self-selected sample. In doing so, it explores possible drivers of those inequities exacerbated by the COVID-19 pandemic that future studies might measure or document at greater depth and breadth.

## Contributions to knowledge

What does this study add to existing knowledge?

- Despite growing recognition of the disproportionate impact of COVID-19 on women, and of the multiple effects on healthcare workers, there is little gender-based analysis of the experiences of women physicians. While an increasing number of studies on health worker well-being during COVID-19 demonstrate higher rates of burnout, anxiety, and financial loss among women, compared to men, they provide little analysis of the determinants of these inequities. This paper offers unique qualitative evidence of experiences of women physicians during the initial COVID-19 response, illuminating how the pandemic has exacerbated underlying drivers of gender inequities within the profession and the need to mitigate these.
  What are the key implications for public health interventions, practice or policy?
- There is a need for greater representation of women within medical and health system leadership, and recognition of women’s crucial contributions at home and work.
- Specific strategies must be developed to provide support to those providing unpaid care work and emotional labour alongside medical expertise. These might include improved access to childcare and mental health resources, among other strategies.
- Without such structural changes, there is a real risk of disproportionate levels of COVID-19-related burnout among women physicians.

## Data Availability

Tables of data organized by theme available upon request.

## Code availability

Coding available upon request.
